# Corneal Stromal Filler Injection as a Novel Approach to Correct Presbyopia—An *Ex Vivo* Pilot Study

**DOI:** 10.1167/tvst.9.7.30

**Published:** 2020-06-25

**Authors:** Stefan Kassumeh, Jannik K. Luther, Christian M. Wertheimer, Katharina Brandt, Merle S. Schenk, Siegfried G. Priglinger, Andreas Wartak, Gabriela Apiou-Sbirlea, R. Rox Anderson, Reginald Birngruber

**Affiliations:** 1 Wellman Center for Photomedicine, Massachusetts General Hospital, Harvard Medical School, Boston, MA, USA; 2 Department of Ophthalmology, University Hospital, LMU Munich, Munich, Germany; 3 Institute of Biomedical Optics, University of Luebeck, Luebeck, Germany

**Keywords:** presbyopia correction, corneal filler, refractive surgery, femtosecond laser, hyaluronic acid, bifocality

## Abstract

**Purpose:**

To evaluate the *ex vivo* feasibility of corneal stromal filler injection to create bifocality to correct presbyopia by flattening the central posterior corneal surface and thus increase refractive power.

**Methods:**

Femtosecond laser-assisted corneal stromal pockets of varying diameters close to the posterior corneal curvature were cut into rabbit eyes *ex vivo*. Subsequently, hyaluronic acid was injected to flatten the central posterior curvature. Refractive parameters were determined using perioperatively acquired three-dimensional optical coherence tomography (OCT) scans. Using micrometer-resolution OCT, corneal endothelial cell morphology and density were evaluated.

**Results:**

Following filler injection into the corneal stromal pockets, a fair volume-dependent increase of central refractive power up to 4 diopters (dpt) was observed. Unremarkable refractive changes of the peripheral posterior (3 mm, 0.20 ± 0.11 dpt; 2 mm, 0.11 ± 0.10 dpt) and the anterior corneal curvature (3 mm, 0.20 ± 0.34 dpt; 2 mm, 0.33 ± 0.31 dpt) occurred. Only negligible changes in astigmatism were observed. Different sizes of optical zones could be established. Furthermore, no alterations of corneal endothelial morphology or endothelial cell density (2831 ± 356 cells/mm^2^ vs. 2734 ± 292 cells/mm^2^; *P* = 0.552) due to the adjacent laser treatment were observed.

**Conclusions:**

The *ex vivo* investigations proved the principle of injecting a filler material into femtosecond laser-created corneal stromal pockets close to the posterior corneal curvature as an efficacious, individually adjustable, and novel approach to correct presbyopia without ablating corneal tissue.

**Translational Relevance:**

Due to the aging population worldwide, presbyopia is an increasing problem; thus, our study may encourage further exploration to extend the treatment spectrum of clinically used femtosecond laser systems to correct presbyopia.

## Introduction

Presbyopia is defined as the loss of accommodative power, leading to difficulties when reading or focusing on objects nearby. By the age of 50, the capability of the eye to increase the refractive power settles at around 0.5 diopters (dpt), compared to 7 to 10 dpt in the first two decades of life. Current studies estimate that presbyopia affects nearly 25% of the world's population.^1^ Patients experience a gradual onset of blurred near vision as one of their first symptoms. The underlying mechanism resulting in the loss of accommodative ability is still poorly understood and inspires controversy among ophthalmologists. Commonly, either lenticular or extralenticular alterations are considered as the main cause, mostly due to a decrease of tissue elasticity.^2^^,^^3^

Non-invasive correction modalities for presbyopia range from a pair of reading glasses and progressive lenses to contact lenses. To date, however, presbyopic patients have indicated a growing desire to live a life without glasses. This desire has led to the development of a variety of surgical methods to correct near-vision problems. Treatment options include laser refractive procedures, corneal inlays, and the implantation of an intraocular lens (IOL). Any of these can be used to achieve monovision or multifocality,^4^ but all of them treat the loss of accommodation temporarily and symptomatically while the presbyopia progresses. To address that issue, accommodative IOLs consisting of multiple zones were proposed to imitate physiological accommodation to a certain extent.^5^

Laser refractive surgeries, such as multifocality-inducing presbyLASIK (laser in situ keratomileusis) and multifocality-inducing photorefractive keratectomy (PRK), are common procedures utilizing excimer laser ablation to reshape the cornea in a way to achieve near and far vision simultaneously.^6^ Many studies on presbyLASIK have reported reasonable results for uncorrected near visual acuity (UNVA) and uncorrected distance visual acuity (UDVA), mainly in hyperopic presbyopic patients.^7^^,^^8^ However, in addition to glare and halos,^9^ a decrease of best-corrected visual acuity and contrast sensitivity can occur in up to 25% of patients following surgery.^10^

Corneal inlays can either make use of the so-called pinhole effect to increase the depth of focus (Kamra inlay)^11^ or create multifocality by changing the refractive index of the cornea (refractive corneal inlays, such as the Flexivue Microlens). Studies on corneal inlays have revealed an improvement in UNVA,^12^^,^^13^ but complications such as a decline in UDVA,^12^ dry eyes, glare, and halos have been reported^14^ and in turn may lead to serious patient discomfort.

To correct presbyopia when patients are not eligible for laser refractive surgeries, monovision, multifocal, extended depth of focus, or accommodative IOLs can be used. All of these can improve binocular UNVA while keeping binocular UDVA unaffected.^15^ Still, the invasive character of the intervention cannot be overlooked, as it may lead to serious complications, including increased intraocular pressure (IOP) and retinal detachment. In addition, visual disturbances may arise, mainly in patients with multifocal IOLs.^16^

A previous *ex vivo* study showed that corneal filler injection is feasible for correcting hyperopia.^17^ The following *ex vivo* pilot study explores the principle of corneal filler injection to establish corneal bifocality to correct presbyopia in an individual and adjustable manner.

## Methods

### Principle of Corneal Bifocality to Correct Presbyopia

**Figure 1.**
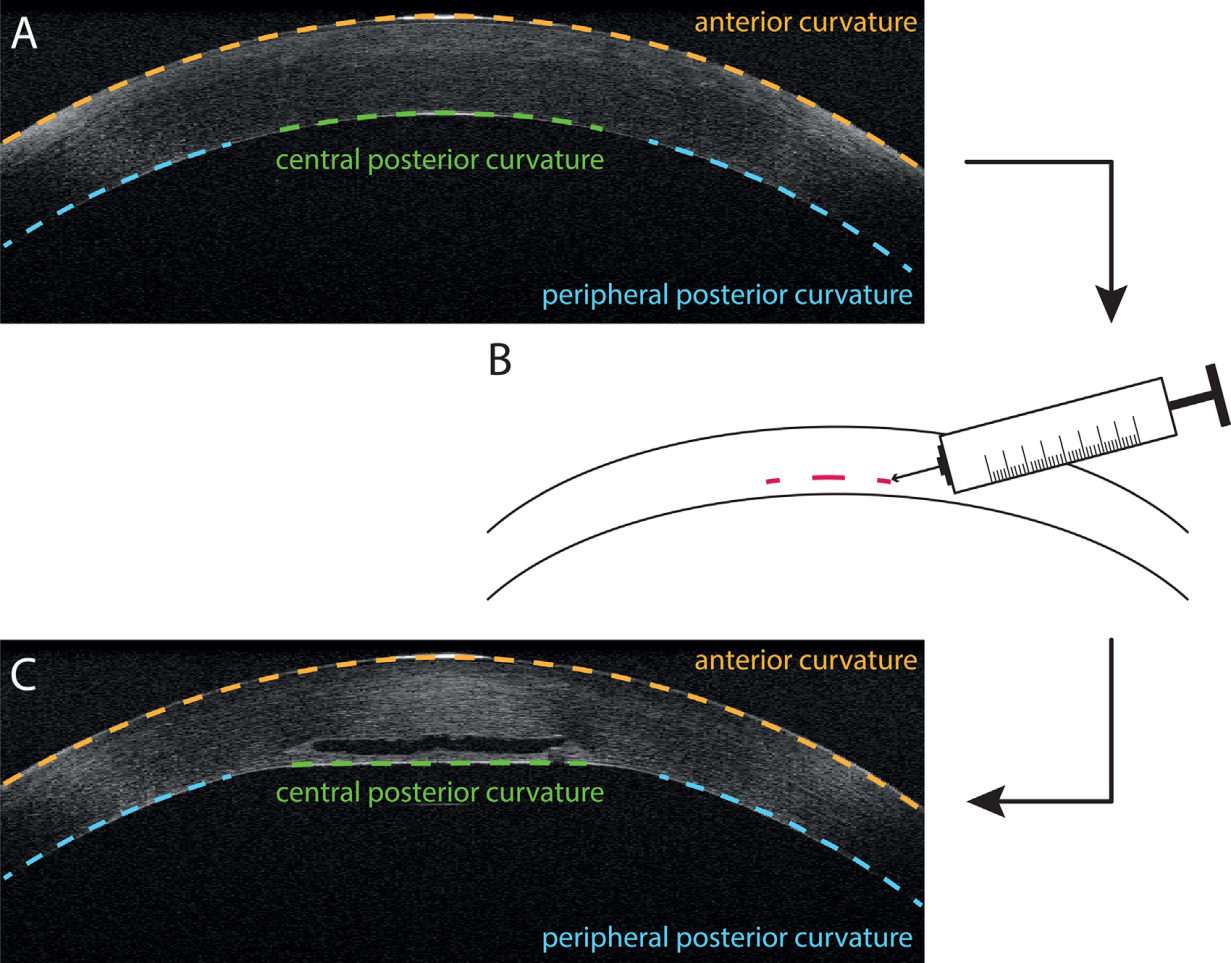
Correction of presbyopia. (A) Representative central OCT B-scan prior to intervention. (B) Filler was injected into a small, deep stromal pocket, close to the posterior corneal curvature. (C) Injected filler led to a flattening of the central posterior corneal curvature.

The aim of our correction methodology was to create bifocality by flattening the central posterior surface of the cornea to create a central zone for near vision. The peripheral zone should remain unchanged for far vision. Stromal pockets with a small diameter and located close to the posterior corneal surface were cut using a state-of-the-art ophthalmologic femtosecond laser system (Visumax; Carl Zeiss Meditec, Jena, Germany). The pockets were filled with hyaluronic acid as filler material. The subsequent flattening of the central posterior curvature resulted in an increase in central refractive power (Fig. 1). We proposed that the anterior corneal curvature and peripheral posterior curvature would remain unaffected by the procedure.

### Corneal Tissue Preparation

New Zealand White rabbit cadaver eyes were obtained from Pel-Freez Biologicals (Rogers, AR) and processed within 24 hours post mortem. To dehydrate the corneas, they were kept in phosphate-buffered saline (PBS; Thermo Fisher Scientific, Waltham, MA) supplemented with 20% w/v dextran (MilliporeSigma, Burlington, MA) for at least 1 hour. Subsequently, whole eyeballs were mounted on a custom-designed eye holder that allowed precise alignment. Furthermore, a water column was attached to maintain a physiologic IOP of approximately 20 mm Hg. The corneas were screened macroscopically, and optical coherence tomography (OCT) was used to sort out damaged and unphysiologically shaped specimens.

### Corneal Filler Material

We used a biocompatible and well-known filler material, hyaluronic acid from *Streptococcus equi* (molecular weight, 2.0–2.4 MDa; MilliporeSigma). A 1% w/v solution of hyaluronic acid sodium salt in PBS was kept at 4°C until utilization. The specifications are similar to those for hyaluronic acids used intraocularly, such as Healon Pro or Healon GV Pro (Johnson & Johnson, Inc., New Brunswick, NJ) or Naluron 1.4 (HumanOptics, Erlangen, Germany), which have molecular weights ranging from 1.1 to 3.0 MDa and hyaluronic acid concentrations between 1% and 1.8% w/v.

### Presbyopia Correction Procedure

Following mounting of the eyeball in an eye holder, a preoperative three-dimensional (3D) OCT image was acquired to analyze the corneal profile as described below. Furthermore, the pachymetry was measured manually as the shortest distance from the corneal apex to the posterior corneal surface in a central OCT B-scan using ThorImage OCT software (Thorlabs Inc., Newton, NJ), to adapt the cutting depth. Dextran residues were gently removed with PBS-moistened K-Sponge Spears (Katena Products, Inc., Parsippany, NJ). A clinical, ophthalmic femtosecond laser system (Visumax; Carl Zeiss Meditec) was used to cut circular stromal pockets with a diameter of either 2 mm or 3 mm. Subsequent to precise alignment, the rabbit eyeball was docked to the human interface (size S) and locked by activating the vacuum suction. To cut the stromal pocket, a sham anterior lamellar keratoplasty was performed using a spot distance and line separation of 2 µm and a pulse energy of 140 nJ. Based on the previous thickness measurements, pocket depths were cut in at a distance of 60 to 300 µm to the posterior corneal surface. After the lamellar cut was finished, the laser procedure was manually terminated. No automatic side cut was performed. Subsequently, under the Visumax built-in microscope, a tunnel beginning close to the limbus and ending at the edge of the pocket was created using a curved 29-gauge insulin syringe and needle (PZN 04400156, U-100 insulin syringe; Becton, Dickinson and Company, Franklin Lakes, NJ). Different volumes of filler material were injected using a 29-gauge blunt-ended insulin needle (PZN 04400156; Becton, Dickinson and Company) that was directly attached to the lumen of a 28-gauge mouse femoral vein catheter (SAI Infusion Technologies, Lake Villa, IL). Only the tip of the femoral vein catheter was inserted into the limbal corneal incision. Air bubbles in the corneal pocket were removed by gentle injection and retrieval of the filler material. To analyze the postoperative changes of refractive power, 3D OCT images were acquired for different volumes of injected filler material in each cornea. During the entire procedure, the cornea was kept moistened by regularly applying PBS (Thermo Fisher Scientific) with a K-Sponge Spear (Katena Products).

### Morphological Investigations of Corneal Endothelium

To investigate whether the impact of the femtosecond laser cut close to the corneal endothelium could affect corneal endothelial cell (CEC) morphology, en-face OCT images of the posterior corneal surface and the corneal endothelium were acquired pre- and postoperatively, using micrometer-resolution OCT (µOCT) as concisely described below. In addition to morphological assessment, the endothelial cell density (ECD) was calculated by measuring the cell area of 25 corneal endothelial cells pre- and postoperatively using ImageJ 1.52p software (National Institutes of Health, Bethesda, MD).

### OCT Systems

To acquire 3D OCT images for corneal profile analysis perioperatively, we used a Thorlabs TEL320C1 spectral-domain OCT (SD-OCT) system with a central wavelength of 1300 nm. The axial resolution in air was 5.5 µm, the maximal lateral resolution was 13 µm, and the A-scan rate was up to 76 kHz. To further reduce image distortion, calibration of the OCT was conducted using the calibration chart provided by the manufacturer and in accordance with the manufacturer's recommendations. The field of view (FoV) for C-scans was 3.15 × 8 × 8 mm (Z × X × Y). The corneal apex was aligned centrally in the XY view.

With some modifications, an experimental SD-µOCT setup as described before^18^ was used for bench-top imaging of the endothelial cell morphology. In short, the system was operated at 800 ± 150 nm with an illumination power at the sample of 20 mW. Volumetric and cross-sectional scans were acquired at a 70-kHz A-scan rate of 500 × 500 lateral pixels and a FoV of 500 µm × 500 µm. The axial and transverse resolutions of the setup were ∼1 µm and ∼2 µm in tissue, respectively.

### Image Post-Processing and Corneal Refraction Analysis

The raw OCT data were further processed in MATLAB (The MathWorks, Inc., Natick, MA). Briefly, all corneal surfaces were selected semi-automatically based on thresholding. The total anterior corneal profile was defined as the central circular area around the apex with a diameter of 6.2 mm (Fig. 1A). The central posterior profile was determined as the central circular area within a diameter of either 1.8 mm (2-mm pocket) or 2.8 mm (3-mm pocket) around the apex. For peripheral posterior curvature, the annular area between the central area and a diameter of 6.2 mm was considered (Fig. 1A). Fitting of every surface was conducted by a least-squares spherical fit (provided by Yury Petrov, Oculus VR, Menlo Park, CA) followed by a toroidal fit. Using the radii of the fitted sphere and torus, the spherical and cylindrical refractive power (spherical equivalent, SE) were determined.

**Figure 2.**
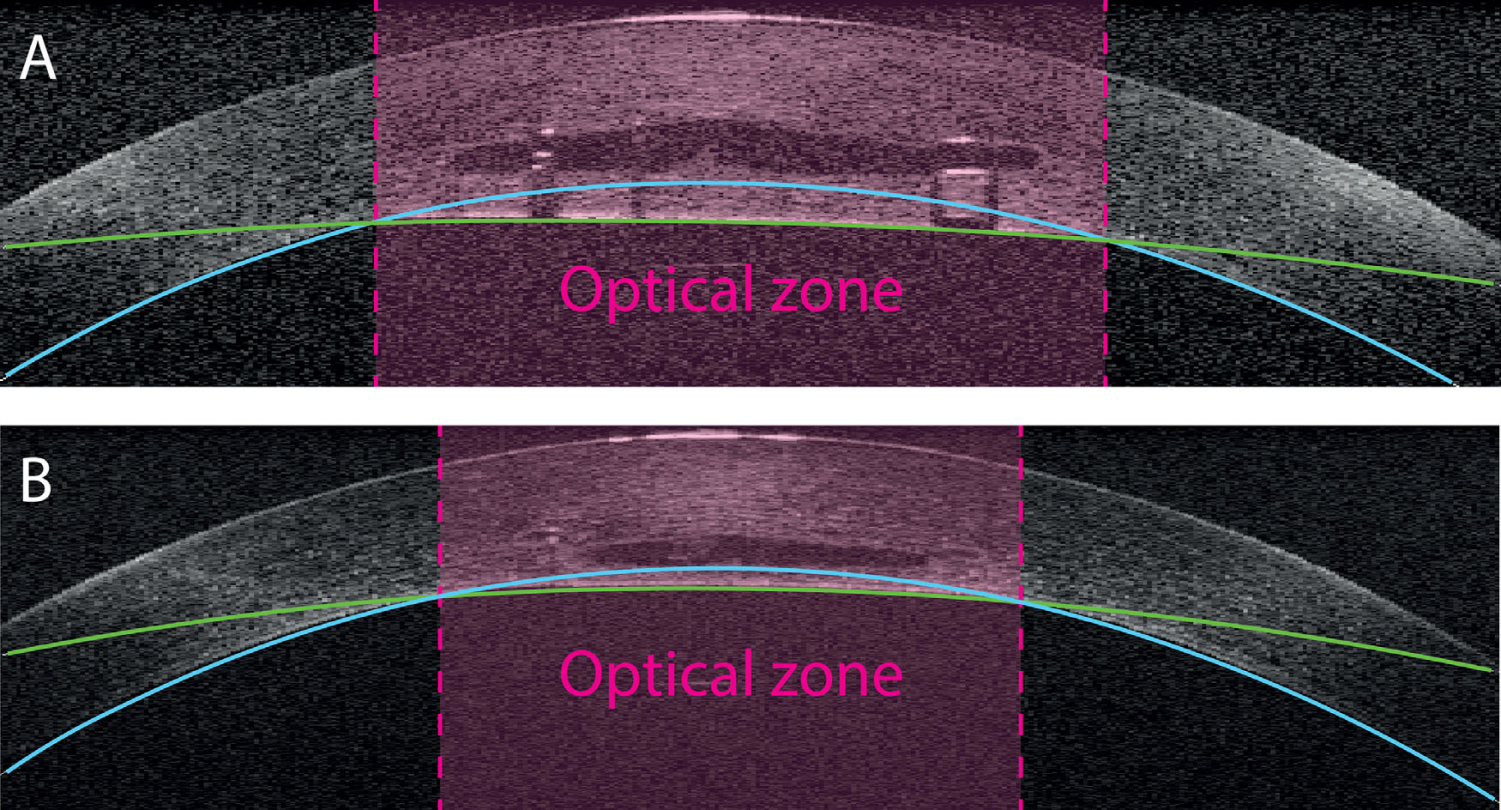
Optical zone. The optical zone was defined as the area where the fits of the central (*green*) and peripheral (*blue*) posterior corneal curvatures intersected. It reflects the actual area treated by the corneal filler injection. Optical zones for the (A) 3-mm pocket diameter and (B) 2-mm pocket diameter.

To calculate the filler volume, voxel volumes between the anterior and posterior surface of the filler pocket were added up. The maximal thickness of the cornea and the thickness of the anterior and posterior lamella were measured as the distance between the two spherical fits of the related filler contours. The optical zone was defined as the cross-sectional circular area and the resulting radius between the spherical fits of the central and peripheral posterior corneal curvature (Fig. 2).

### Experimental Groups, Data Management, and Statistical Analysis

In total, 57 rabbit cadaver eyes were used, of which 36 received pockets with a diameter of 3 mm and 21 pockets with a diameter of 2 mm. Depths were chosen at a distance from the posterior corneal surface of 80 to 300 µm for 3-mm pockets and 60 to 200 µm for 2-mm pockets. Data management and calculations were performed using Excel (Microsoft Corp., Redmond, WA) and MATLAB. To compare two independent experimental groups, a Student's *t*-test was conducted. *P* values less than 0.05 were considered to be statistically significant. Graphs and regressions (*r*^2^ and Pearson) were plotted with Prism 8 (GraphPad Software, San Diego, CA), and figures were prepared with Illustrator 2020 (Adobe, Inc., San Jose, CA).

## Results

### Increased Central Posterior Refractive Power Is Volume Dependent

**Figure 3.**
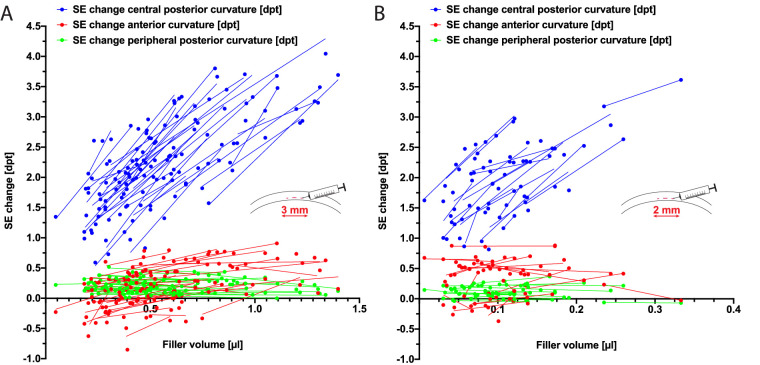
Volume-dependent increase of SE of the posterior corneal curvature. Up to five increasing filler volumes (*dots*), injected into a single cornea, led to decent linear refractive power gains (*lines*) by flattening the posterior corneal curvature. Unremarkable refractive changes of the anterior and peripheral posterior curvature were observed. Shown are the corrected corneas with pocket diameters of (A) 3 mm (n = 36) and (B) 2 mm (n = 21).

In every single cornea, up to five different amounts of filler material were injected and the refractive power of the different corneal curvatures was measured. Overall, volumes ranging from 0.05 to 1.4 µl (3-mm pocket) and 0.01 to 0.33 µl (2-mm pocket) were determined postoperatively using 3D OCT images. Figure 3 shows the refractive power change of the different corneal surfaces depending on the injected filler volume. For every cornea individual offset, rising filler volumes led to a reasonable linear gain in refractive power of the central posterior corneal curvature (*r*^2^ = 0.90 ± 0.13 for pockets with a 3-mm diameter and *r*^2^ = 0.84 ± 0.19 for pockets with a 2-mm diameter). By flattening the central posterior corneal curvature, the refractive power could be increased by up to 4.1 dpt (Fig. 3A) and 3.6 dpt (Fig. 3B) in pockets with diameters of 3 mm and 2 mm, respectively. The mean individual offset for the lowest injected filler volume in a single cornea with a pocket diameter of 3 mm was 1.4 ± 0.5 dpt (Fig. 3A), and the offset was 1.4 ± 0.5 dpt for 2-mm pocket diameters (Fig. 3B).

### Changes in Refractive Power of Anterior and Peripheral Posterior Curvature

Figure 3 indicates the refractive power changes in the anterior and peripheral posterior corneal curvature. Comparing the pre- and postoperative refractive power of the anterior curvature, we observed a non-significant change of 0.2 ± 0.3 dpt (*P* = 0.362) for 3-mm pockets (Fig. 3A) and 0.3 ± 0.3 dpt (*P* = 0.166) for 2-mm pockets (Fig. 3B). In addition, we were not able to observe a remarkable change in refractive power of the peripheral posterior corneal curvature, whether in 3-mm pockets (0.2 ± 0.1 dpt) or in 2-mm pockets (0.1 ± 0.1 dpt).

### Depth-Dependent Maximum Refractive Change

**Figure 4.**
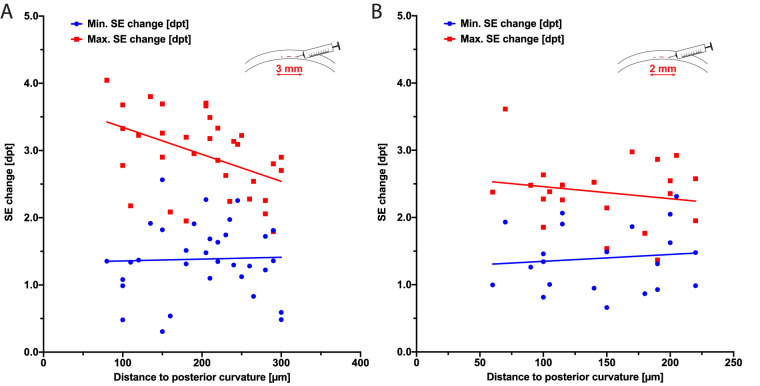
Depth dependence of refractive change. The minimal and maximal refractive changes induced by corneal filler injection were compared to the postoperatively measured distance of the pocket to the posterior corneal surface. (A) Pockets with a diameter of 3 mm (n = 36), and (B) pockets with a diameter of 2 mm (n = 21).

We measured the refractive power changes due to injection of minimally and maximally possible filler volumes. In the 3-mm pockets, a tendency of lower maximal refractive power changes with increasing distance to the posterior corneal curvature could be observed (Fig. 4A). At a distance of 80 µm to the posterior corneal curvature, a maximum refractive change of 4.1 dpt could be observed; at a distance of 300 µm, a change of 2.9 dpt was observed. Interestingly, there was no similar effect in the 2-mm-diameter pockets (Fig. 4B).

### Optical Zone Depending on the Pocket Diameter

Figure 2 shows the optical zone on representative cross-sectional OCT scans through the corneal apex for both pocket diameters. The mean optical zones in corneas with either a 3-mm pocket or a 2-mm pocket were 3.56 ± 0.21 mm (n = 36) and 2.81 ± 0.31 mm (n = 21), respectively. Injecting different volumes of filler material did not change the optical zone (data not shown).

### Changes of Astigmatism

The radii derived from the toroidal fits of the anterior corneal curvature were used to calculate the corneal astigmatism. The astigmatic axis was defined as the axis of the steep radius of the related toroidal fit. Among all eyes, we could observe only minor changes of astigmatic power and axis. For treated corneas with a 3-mm pocket, a median refractive change of –0.2 ± 0.5 dpt and axis change of –3.8 ± 7.8° was measured. Similar changes were observed when corneas were treated with a 2-mm pocket (–0.1 ± 0.4 dpt and 4.5 ± 7.2°).

### Morphology of Corneal Endothelial Cells and Endothelial Cell Density

**Figure 5.**
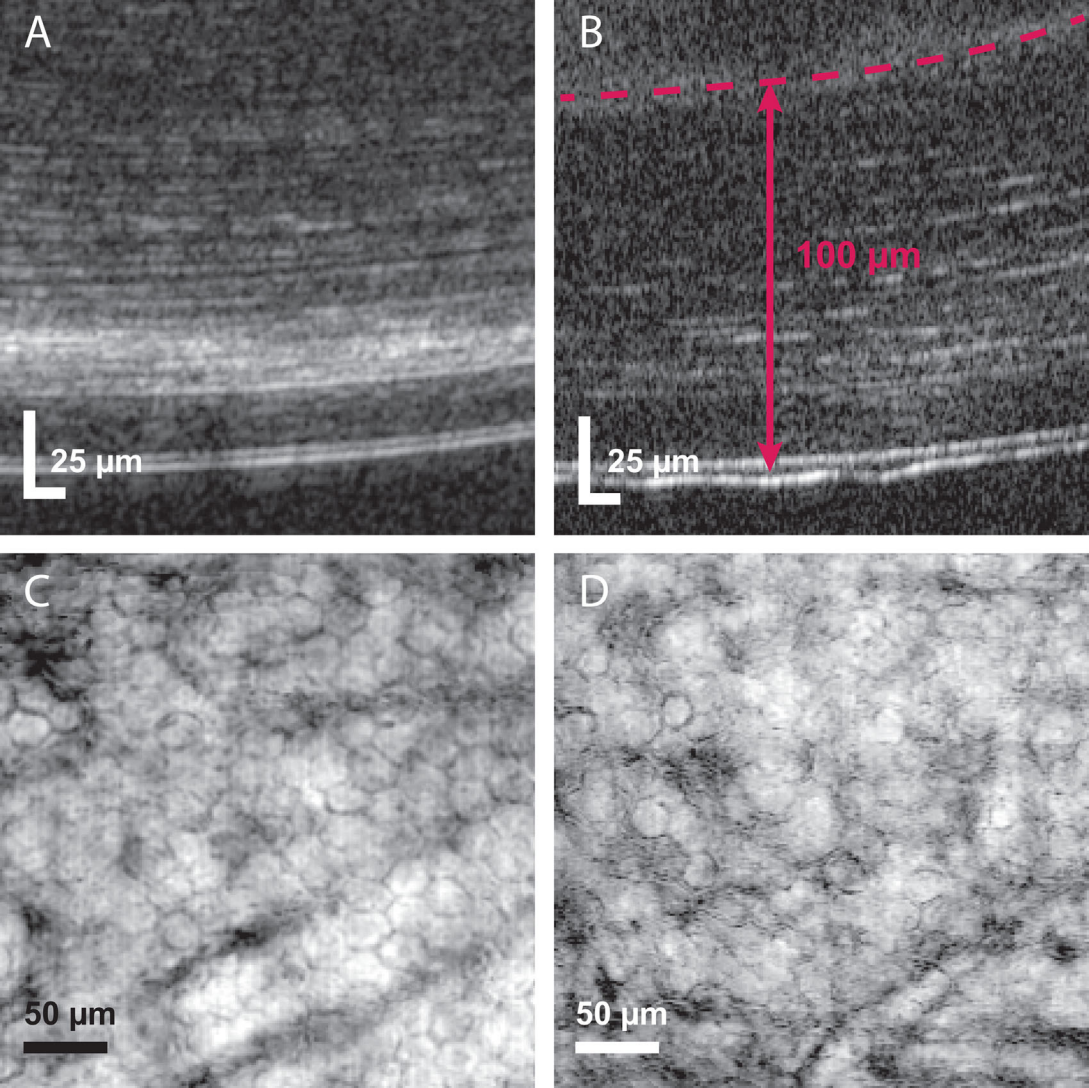
Unaffected corneal endothelial cell morphology. (A) Single representative µOCT B-scan of the untreated posterior corneal stroma and corneal endothelium. (B) Single representative µOCT B-scan following corneal filler injection into the pocket (*red dashed line* corresponds to lower pocket boundary). Distance to the posterior corneal curvature was ∼100 µm. (C, D) Pre- and postoperative en face µOCT projections of corneal endothelial cells.

When comparing corneal endothelial cell morphology pre- and postoperatively, no manifest morphological alterations could be observed in the µOCT image data. The corneal pocket of the depicted cornea was at an actual postoperatively measured distance to the posterior corneal curvature of 100 µm (Fig. 5B). In en face µOCT images, CECs still showed their typical hexagonal arrangement and dense cell–cell contacts (Figs. 5C, 5D). Furthermore, the measurement of the cell area pre- and postoperatively did not reveal a significant difference in endothelial cell density (2831 ± 356 cells/mm^2^ vs. 2734 ± 292 cells/mm^2^; *P* = 0.552).

## Discussion

Based on the following four findings, our results suggest that corneal filler injection into deep stromal pockets, close to the posterior corneal surface, is a feasible novel approach to inducing bifocality to correct presbyopia *ex vivo*: (1) nearly linear, volume-dependent increase of central refractive power up to 4 dpt; (2) negligible refractive power changes of approximately 0.2 dpt of the peripheral posterior and of less than 0.3 dpt of the anterior corneal curvature, thus creating bifocality; (3) minor changes in astigmatism, less than –0.2 dpt of refractive power and 4.5° of axis; and (4) no morphological alterations of corneal endothelial and no significant decrease in corneal endothelial cell density due to the adjacent laser treatment and filler injection.

Presbyopia correction by injecting a viscous filler material into deep stromal pockets may, in the future, become a competitor within the landscape of state-of-the-art presbyopia correction procedures, such as presbyLASIK or corneal inlays. Such interventions make future adjustments more difficult, especially ablative procedures, as they remove corneal tissue.^6^ Previous studies on a multifocal ablative approach to correct presbyopia revealed a retreatment rate of approximately 20%.^19^^,^^20^ With regard to patient dissatisfaction or complications with corneal inlays and presbyopia-correcting IOLs, up to 18% of the patients have required an explantation of the implant, requiring yet another invasive surgery that could result in further complications.^21^^–^^23^ Our results indicate a volume-dependent, linear increase of the central refractive power. This approach, in theory, offers correction that can be adjusted to suit the individual's need; for example, patients at a younger age suffering from mild presbyopia might only need a minor correction of around +1.0 dpt. As presbyopia progresses, additional filler material could be added to the pocket to increase the refractive correction by up to +4 dpt, depending on the actual need. Furthermore, the tunnel used to inject filler material is smaller than the incision used in the small incision lenticule extraction procedure but is linked to a long tunnel. No serious postoperative pain is expected. If retreatment is necessary, the tunnel could be reopened, similar to reopening of the LASIK flap.^24^^,^^25^ In our case, the tunnel might easily be dissected with a blunt needle, and the amount of filler material could be readjusted or removed completely in case of complications.

The principle of bi- or multifocality to compensate for presbyopia is commonly applied for surgical treatment of the condition. In general, this approach produces pupillary areas of varying optical power to allow simultaneous sharp vision of far objects and those nearby,^26^ similar to, for example, presbyLASIK. The corneal filler approach follows the principle of bifocality, but modifications are performed on the inner, less sensitive surface of the cornea. Furthermore, no corneal tissue is ablated. By using two different pocket diameters, variation of optical zones (between approximately 2.8 and 3.6 mm) was achieved, offering adaptability to individual pupil dynamics. We have demonstrated the feasibility of both pocket sizes and pockets with varying depths, although a long journey toward achieving a clinical approach remains. Further studies must investigate the most feasible, biocompatible, and suitable dimensions of the pocket. In addition, current femtosecond laser platforms used in ophthalmology are not capable of precisely cutting pockets with such small diameters and at the depths necessary. For this reason, technical and software adjustments must be made before approaching a human *in vivo* study.

Corneal endothelial water pumps are essential to maintaining the balanced hydration status of the cornea and thus its transparency. As the corneal endothelial cell count decreases with age,^27^ additional, iatrogenic damage is to be avoided. Our µOCT imaging results before and after femtosecond laser-mediated pocket creation showed no distinct corneal endothelial cell damage and no significant decrease in corneal endothelial cell density. Previous studies investigating corneal endothelial cell density in healthy rabbits using specular microscopy revealed a mean ECD of 2919 ± 177 cells/mm^2^,^28^ which matches our postoperative measurements. Further, our findings are in line with different studies on corneal endothelial damage during graft preparation for Descemet stripping automated endothelial keratoplasty (DSAEK). Liu et al.^29^ observed only a minor loss of endothelial cell density in femtosecond laser-assisted cuts at a distance of 90 µm from the endothelium. Similar observations were made by Phillips et al.^30^ Preparing DSAEK grafts with a remaining posterior corneal thickness of approximately 60 µm led to a minimal, non-significant damage of corneal endothelial cells. Still, further *in vivo* studies should be conducted to address any concerns related to femtosecond laser safety.

Hyaluronic acid is naturally present in the cornea and commonly used during cataract, refractive, and glaucoma surgery. Extensive research was performed on the biocompatibility of hyaluronic acid, as well as its pharmacological effects.^31^ Due to its high molecular weight (>2 MDa), diffusion into the corneal stroma seems unlikely. Two previous case reports found that hyaluronic acid accidentally injected into the corneal stroma remained stable, did not show any resorption tendency, and did not cause corneal scarring for at least 3 years.^32^^,^^33^ Therefore, in terms of stability, hyaluronic acid seems to be a feasible filler material. However, long-term *in vivo* investigations should be conducted to determine whether a secondary inflammatory response occurs that may lead to corneal haze or keratopathy.

Because a major goal for ophthalmologists when correcting presbyopia is patient satisfaction, reliable predictability of the refractive outcome is of utmost importance. The results presented here clearly indicate a filler volume-dependent refractive increase. Furthermore, at least in 3-mm pockets, we could observe a depth-dependent maximum refractive change; however, an individual and varying offset for the minimally possible refractive change was measured. As some linear fits intersect with the origin, it can be assumed that, under optimal experimental conditions, a titration of the refractive change may be achievable. Additionally, when considering the maximum and minimum refractive change in 2-mm pockets, we did not observe decreasing maximum correction with greater distance to the posterior corneal curvature compared to the 3-mm pockets. We did encounter some difficulty in establishing the filler tunnel manually and in injecting and analyzing submicroliter amounts of filler material; thus, we may not have detected small refractive changes that could explain the offset seen for the SE change, as well as the missing depth-dependent maximum refractive change, for 2-mm pockets. We encourage further investigations using femtosecond laser-created tunnels and submicroliter injection systems to demonstrate the practicability and titratability of the filler-mediated presbyopia correction prior to evaluating a precise volume dependency of refractive changes *in vivo*.

In summary, we have presented an early *ex vivo* safety and efficacy investigation of corneal filler injection as a novel treatment option for presbyopia. Advantages include being able to adjust the procedure for each individual without having to remove corneal tissue, in addition to the minimally invasive character of the procedure. We believe that this technique warrants further exploration as a promising surgical modality for the correction of presbyopia.
